# Omicron SARS-CoV-2 Variant Spike Protein Shows an Increased Affinity to the Human ACE2 Receptor: An In Silico Analysis

**DOI:** 10.3390/pathogens11010045

**Published:** 2021-12-31

**Authors:** Joseph Thomas Ortega, Beata Jastrzebska, Hector Rafael Rangel

**Affiliations:** 1 Cleveland Center for Membrane and Structural Biology, Department of Pharmacology, School of Medicine, Case Western Reserve University, 10900 Euclid Ave, Cleveland, OH 44106-4965, USA; jto22@case.edu; 2 Laboratorio de Virología Molecular, Centro de Microbiología y Biología Celular, Instituto Venezolano de Investigaciones Científicas, Apdo 20632, Caracas 1020A, Venezuela

**Keywords:** omicron, SARS-CoV-2, spike, variants, binding affinity

## Abstract

The rise of SARS-CoV-2 variants, with changes that could be related to an increased virus pathogenicity, have received the interest of the scientific and medical community. In this study, we evaluated the changes that occurred in the viral spike of the SARS-CoV-2 Omicron variant and whether these changes modulate the interactions with the angiotensin-converting enzyme 2 (ACE2) host receptor. The mutations associated with the Omicron variant were retrieved from the GISAID and covariants.org databases, and a structural model was built using the SWISS-Model server. The interaction between the spike and the human ACE2 was evaluated using two different docking software, Zdock and Haddock. We found that the binding free energy was lower for the Omicron variant as compared to the WT spike. In addition, the Omicron spike protein showed an increased number of electrostatic interactions with ACE2 than the WT spike, especially the interactions related to charged residues. This study contributes to a better understanding of the changes in the interaction between the Omicron spike and the human host ACE2 receptor.

## 1. Introduction

The viral infection related to SARS-CoV-2 in humans is a recent evolutionary event. However, the occurrence of genetic mutations is increasing continuously while the virus is disseminated worldwide, allowing the identification of several viral variants that are actively circulating in the population [1]. These mutations, depending on the location and the type, can produce changes in the viral fitness and/or pathogenicity [2,3]. The spike protein, which plays a pivotal role in the early events in viral replication, is a hot spot for such changes [4,5]. The viral spike participates in the interaction with the host receptor angiotensin-converting enzyme 2 (ACE2), which is highly expressed in human airway cells [6]. The wild-type (WT) SARS-CoV-2 spike already showed changes that likely increased the efficiency of the virus–ACE2 receptor interactions as compared to SARS-CoV [7]. Major changes were noted in the region located within the residues 493 and 505 [7]. Thus, the SARS-CoV-2 viral variants could have a different transmissibility and response to neutralizing antibodies [8,9,10]. The mutations found in the different viral variants can occur in any part of the genome. However, some mutations that arose within the receptor-binding domain (RBD) received the most interest from researchers and medical groups due to its importance in host recognition and possibly changes in viral fitness [8,11]. One of the first variants reported and widely distributed worldwide was the Alpha variant (B.1.1.7 linage) [12]. Later, the other variants, including Beta, Delta, and Mu, were found in the population (https://www.cdc.gov/coronavirus/2019-ncov/science/science-briefs/scientific-brief-emerging-variants.html (accessed on 2 December 2021)). These variants contain several mutations in their genome that could modulate viral infectivity. In this study, we focused our interest on the mutations that occurred in the spike region interacting with ACE2, which could play a direct role in the modulation of the spike–ACE2 binding pattern. The Alpha variant has a non-synonymous substitution of N501Y, while the Beta variant, as well as Gamma and Mu variants additionally contain the E484K mutation (Table 1). Previous reports showed that these mutations most likely increase the binding affinity of the spike to ACE2 as compared to the WT spike, without affecting its overall structure [13,14,15]. These mutations are located primarily in the protein loops, which due to their high flexibility could likely tolerate the conformational changes associated with the residue substitution. Interestingly, most of the single residue mutants exhibit a lower binding free energy, thus higher binding affinity to the host receptor in comparison to the WT variant [15]. The increased affinity of these viral variants to the host receptor could be explained by an increase in the number of hydrogen bonds and pi–pi interactions, as well as the change in charged residue interactions. The information gained from the analysis of the single residue mutants allowed us to hypothesize that variants carrying multiple mutations in the RBD will exhibit an even higher stabilizing effect on the interaction between the viral spike and the host ACE2 receptor [15]. The recently emerged Omicron variant could prove the correctness of our hypotheses. The Omicron variant carries over 30 mutations in the viral spike, among which eight mutations are located directly in the receptor-binding region of the RBD [16]. These mutations possibly could increase the binding stability of the spike to the ACE2. This variant shows a significant increase in the number of mutations in comparison to the Alpha variant that has one residue mutated in the RBD as well as the Beta and Gamma variants that contain three residues mutated in the RBD (Table 1 and Figure S1).

## 2. Results and Discussion 

To gain a deeper understanding on how these changes in the Omicron spike variant impact its binding affinity to the human host ACE2, we performed a molecular docking analysis using two different approaches, Zdock/Prodigy and Haddock (see Supplementary Materials for details). The obtained results indicated that the mutations that occurred in the RBD of the Omicron variant resulted in an increased number of interactions between the spike and ACE2 receptor, especially, the number of interactions associated with the charged residues was increased (Table 2).

Importantly, it has been previously described that the interactions between the charged residues of the viral spike and the host ACE2 play a major role in the complex stabilization and host selectivity [7,18,19]. These changes in the number and type of interactions were reflected in a decreased binding free energy (Table 2) and a decrease in the electrostatic, van der Waals interactions, and desolvation energies with an overall lower haddock score obtained for the Omicron variant as compared to the WT variant (Table 3).

**Figure 1:**
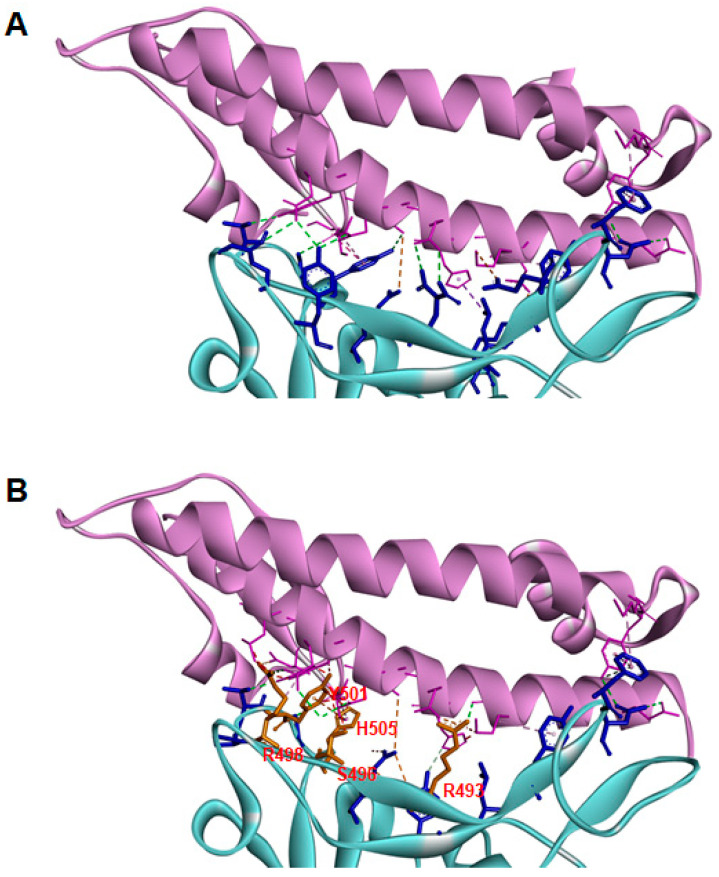
The binding pattern of the Omicron SARS-CoV-2 spike to ACE2 receptor. A close-up view of the interaction complex between the SARS-CoV-2 WT (**A**) or Omicron (**B**) spike and the human ACE2. The results obtained from molecular docking analysis for the interaction between the SARS-CoV-2 spike (blue) and ACE2 (pink) are shown. The residues mutated in the Omicron variant (R493, S496, R498, Y501, H505) that interact directly with the ACE2 are shown in orange. The sequence for the SARS-CoV-2 viral spike protein was retrieved from the Uniprot server (sequence number: P0DTC2) and the homology structural model for the Omicron variant was built by using the tools of the SWISS-MODEL modeling server and the DeepView/Swiss-PdbViewer 4.01 software [20]. ProSA-web and PROCHECK programs were used to validate the quality of the structure [21,22]. Hydrogen atoms were added, and partial charges were assigned for the energy refinement, as described in [23,24]. The crystal structure of the SARS-CoV-2 spike protein bound to the human ACE2 receptor (PDB code: 6M0J) and the structure of the human ACE2 receptor (PDB code: 1R42) were downloaded from the Protein Data Bank. The Omicron variant and the WT spike proteins were evaluated. First, the docking between a ligand (RBD of the WT or Omicron spike protein) and a receptor (ACE2) was performed with Z-Dock software [25], and the obtained complexes were processed and analyzed by using the tools in the PRODIGY software [26]. Furthermore, docking analysis for the Omicron spike protein was assayed using the Haddock server [27].

The main interactions between the viral spike and the host ACE2 for the Omicron variant are shown in Figure 1. The mutated residues in the RBD that directly interact with ACE2 are shown in orange. Interestingly, most of these residues are localized in the region between 493 and 505, which was previously described as important for the evolution between SARS-CoV and SARS-CoV-2 [7]. Furthermore, evaluation of the individual SARS-CoV-2 spike mutations in residue 505 revealed that the change from tyrosine to tryptophan resulted in the lower binding free energy interaction complex. In the Omicron variant, Y505 is substituted by the histidine residue, which resulted in fewer pi–pi interactions than observed for the double aromatic ring present in tryptophan [15]. However, histidine flexibility produces an increase in the number of interactions of the viral spike with other residues of ACE2, including E35 and D355, which could further stabilize the interaction between the spike and the host receptor (Table 4). In addition, other mutated residues, such as R493, Y501, and R498, showed changes in the type and number of interactions with ACE2 that are most likely related to conformational changes occurring in the spike protein, enhancing the overall stability of the spike–ACE2 complex (Table 4).

Thus, this cluster located in the region between residues 498 to 505 could represent an evolutionary region that could be evaluated to predict the binding affinity of the emerging viral variants toward ACE2 in the human host.

**Figure 2:**
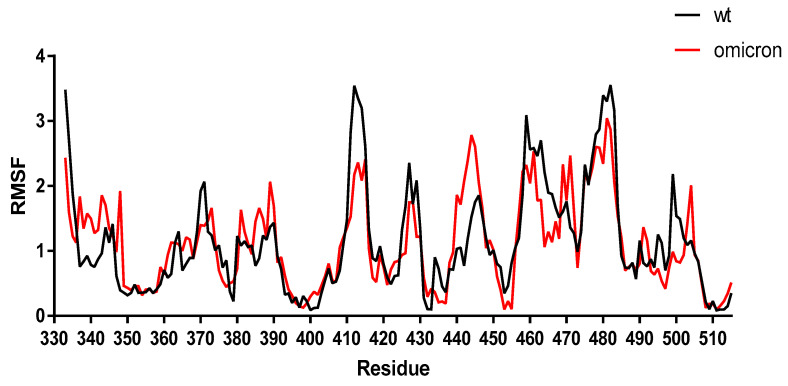
Changes in the pattern of flexibility in the interaction between the spike protein–ACE2 receptor complexes for Omicron and WT. The root-mean-square fluctuation (RMSF) of the protein Cα atoms with the respect to the initial structure for the evaluated spike protein–ACE2 complex was obtained by molecular docking. The spike protein–ACE2 receptor complexes were evaluated using the CABS-flex software [28]. The PDB files were submitted to the CABS-flex server to further assess the stability of the spike protein mutants–ACE2 complexes, and the parameters were adjusted to the default. The MD simulations output data obtained with both software were analyzed according to RMSF. This software enables an efficient modeling procedure for short simulations, being able to produce an analysis of the protein dynamics consistent with the dynamics obtained from 10-nanoseconds MD simulations with the most popular force fields.

To validate further the results obtained by the molecular docking, the stability of the SARS-CoV-2 Omicron spike–ACE2 complex was analyzed using MD simulations and compared to the WT spike–ACE2 complex. The results showed a similar trend of change in the residue fluctuation in the RBD region for both the WT and Omicron spike protein, confirming our results obtained by molecular docking (Figure 2). The median average root-mean-square fluctuation (RMSF) obtained with the CASB-flex software for the WT and Omicron variant was 1.05 ± 0.37 Å and 1.09 ± 0.25 Å, respectively. However, some differences in the fluctuations were noted for the residues 493, 501, and 505 due to the presence of the mutations.

Herein, we discussed the importance of the viral spike for the entry into the cell and focused on the effects of the point mutations on the binding affinity to the host receptor ACE2. However, it is important to stress that the viral spike, despite targeting the host for virus replication, also plays a major role in the infection resolution, by being blocked with the antibodies either generated by the human host defense system upon infection or after vaccination, or by therapeutic monoclonal antibodies [29,30]. Mutations occurring in the viral genome could result in the evolution of so-called ‘escape variants’; some of these variants can show reduced susceptibility to antibodies [31,32]. Though, the previous analysis showed that changes in the spike binding affinity to the human host receptor did not correlate with changes in the response to monoclonal antibodies [15,33,34]. Moreover, some reports elucidated that the host antibodies generated after vaccination with the currently available mRNA vaccine could neutralize the prior circulating variants such as Alpha and Delta [35,36,37,38]. However, a change in neutralization efficiency was observed for variants carrying the E484K mutation [33]. This is related to the antibody recognition site within the S1 region of the spike protein located between the residues 301 and 430 in the main receptor-binding motif [33,39,40]. The Omicron variant carries several mutations situated in this region. Thus, further analyses to assess the efficiency of the immune response elicited by vaccines to this variant are necessary. Importantly, the vaccination efforts need to be boosted up, ensuring broad coverage across the world to prevent the selection of new viral variants. In the current pandemic scenario, the world has observed that new variants can change the SARS-CoV-2 virus distribution dynamics in terms of prevalence in the population. The Delta variant has been the most prevalent before the rise of Omicron. This variant carries several mutations that could affect viral fitness. However, within the RBD of the spike protein there are only two mutated residues, L452R and T478K. These residues do not interact directly with the ACE2 receptor. However, the residue substitution produced a conformational change in the viral spike, enhancing the binding affinity of the virus to the host receptor. On the other hand, the Omicron variant carries at least eight mutations in key residues that interact directly with the host ACE2. The substitutions in the directly interacting residues resulted in stabilization of the viral spike–ACE2 interaction, evidenced by a decrease in the binding free energy found for Omicron as compared to WT by our molecular docking analysis. Thus, sequence analysis evaluating the mutations present in the viral variants within the region interacting with the host receptor ACE2 could enhance our understanding of the changes in the prevalence dynamics in the SARS-CoV-2 infection. Together, the results presented in this study indicate that mutations that emerged in the new SARS-CoV-2 Omicron variant enhance the binding affinity of this variant to the host ACE2 receptor, which likely is an important driver of its increased transmissibility. However, it is important to note that SARS-CoV-2 besides ACE2 could also use other cellular receptors as a secondary binding way to target the host cells [19,41,42,43]. Thus, it cannot be excluded that the changes that emerged in the Omicron viral spike could have an impact on the virus interaction with these secondary cellular receptors, additionally increasing the virus’s ability to reach its target. Nevertheless, further analyses employing the structural, computational, and biochemical approaches are required to gain a deeper understanding of the structural details of the residue rearrangements and changes in the specific virus–host interactions triggering higher transmissibility of the SARS-CoV-2 Omicron variant.

## Figures and Tables

**Table 1:** Mutations occurring in the SARS-CoV-2 main concern variants.

Residue	WT	Alpha	Beta	Gamma	Delta	Omicron	Mu
339	G					D	
371	S					L	
373	S					P	
375	S					F	
417	K		N	T		**N**	
440	N					K	
446	G					S	
452	L				R		
477	S					N	
478	T				K	**K**	
484	E		K	K		**A**	K
493	Q					R	
496	G					S	
498	Q					R	
501	N	Y	Y	Y		**Y**	Y
505	Y					H	

The residues shared between Omicron and the other variants are shown in bold and underlined. The viral variants data were retrieved from the GISAID [17] and covariants.org webservers, on 26 November 2021. WT (B.1), Alpha (B.1.1.7), Beta (B.1.357), Gamma (P.1), Delta (B.1.617.1), Omicron (B.1.529), Mu (B.1.621).

**Table 2:** Predicted interactions for the Omicron SARS-CoV-2 variant obtained with Zdock/Prodigy.

Parameter	WT	Omicron
Binding energy (kcal/mol)	−11.3	−12.6
ICs charged-charged:	4	12
ICs charged-polar:	10	8
ICs charged-apolar:	18	23
ICs polar-polar:	4	4
ICs polar-apolar:	22	18
ICs apolar-apolar:	10	13
Total number of ICs	68	78

ICs—number of interfacial contacts.

**Table 3:** Predicted interactions for the Omicron SARS-CoV-2 variant obtained with HADDOCK.

Parameter	WT	Omicron
HADDOCK score	−109.8 +/− 3.5	−163.8 +/− 4.1
van der Waals energy	−60.3 +/− 2.9	−111.0 +/− 3.8
Electrostatic energy	−148.9 +/− 44.9	−382.8 +/− 42.1
Desolvation energy	−34.2 +/− 9.2	−13.9 +/− 5.2
Buried Surface Area	1778.5 +/− 96.8	2705.1 +/− 35.2
Z-Score	−2.1	−2.1

**Table 4:** The main interactions between the Omicron spike protein and human ACE2 receptor.

ACE2	WT Spike	Omicron Spike
S19	A475	A475 (2)
Q24	A475, G476, S477, F486, N487, Y489	A475, G476, A477, F486, N487, Y489
T27	F456, Y473, A475, Y489	F456, Y473, A475, Y489
P28	N487, Y489	N487, Y489
D30	K417, L455, F456	N417, L455, F456, **R493**
K31	L455, F456, GLU484, Y489, F490, Q493	L455, F456, Y489, F490, **R493**
F32	---	**R493**
H34	K417, Y453, L455, Q493	N417, Y453, L455, **R493**
E35	Q493, R403, Y505	**R493**, R403, **Y501**, **H505**
D38	Y449, G496, Q498	Y449, **S496**, R498, **Y501**
F40	---	**Y501**
Y41	Q498, T500, N501	R498, T500, **Y501**
Q42	V445, G446, G447, Y449, Q498	V445, S446, G447, Y449, R498, **Y501**
L45	V445, Q498, T500, F486	V445, R498, T500, F486
M82	F486	F486
Y83	F486, N487, Y489	F486, N487, Y489
N330	T500	T500, T500
L351	---	R498, **Y501**
G352	---	**Y501**, **H505** (2)
k353	Y495, G496, F497, Q498, T500, N501, G502, Y505	Y4895, **S496**, F497, R498, T500, **Y501**
G354	T500, N501, G502, V503, Y505	T500, **Y501**, G502, V503, **H505**
D355	T500, N501, G502	R498, T500, **Y501**, G502, **H505**
R357	T500	R498, T500
A386	Y505	---
R393	Y505	---

**---**–No interactions present. (**2**) Two different interactions with the same residue. The mutated residues present in the RBD that interact with the ACE2 receptor are shown in bold.

## Data Availability

Data on SARS-CoV-2 variants was retrieved from the GISAID [17] and covariants.org webservers, on 26 November 2021. The sequence for the SARS-CoV-2 viral spike protein was retrieved from the Uniprot server (sequence number: P0DTC2). The crystal structure of the SARS-CoV-2 spike protein bound to the human ACE2 receptor (PDB code: 6M0J) and the structure of the human ACE2 receptor (PDB code: 1R42) were downloaded from the Protein Data Bank.

## Supplementary Materials

The following are available online at https://www.mdpi.com/article/10.3390/pathogens11010045/s1, Figure S1: Alignment of a portion of SARS-CoV2 spike.

## References

[1] Zeyaullah M., AlShahrani A.M., Muzammil K., Ahmad I., Alam S., Khan W.H., Ahmad R. (2021). COVID-19 and SARS-CoV-2 Variants: Current Challenges and Health Concern. Front. Genet..

[2] Sun F., Wang X., Tan S., Dan Y., Lu Y., Zhang J., Xu J., Tan Z., Xiang X., Zhou Y. (2021). SARS-CoV-2 Quasispecies Provides an Advantage Mutation Pool for the Epidemic Variants. Microbiol. Spectr..

[3] Tao K., Tzou P.L., Nouhin J., Gupta R.K., de Oliveira T., Kosakovsky Pond S.L., Fera D., Shafer R.W. (2021). The biological and clinical significance of emerging SARS-CoV-2 variants. Nat. Rev. Genet..

[4] Bobay L.M., O’Donnell A.C., Ochman H. (2020). Recombination events are concentrated in the spike protein region of Betacoronaviruses. PLoS Genet..

[5] Chakraborty S. (2021). Evolutionary and structural analysis elucidates mutations on SARS-CoV2 spike protein with altered human ACE2 binding affinity. Biochem. Biophys. Res. Commun..

[6] Zhang J., Xiao T., Cai Y., Chen B. (2021). Structure of SARS-CoV-2 spike protein. Curr. Opin. Virol..

[7] Ortega J.T., Serrano M.L., Pujol F.H., Rangel H.R. (2020). Role of changes in SARS-CoV-2 spike protein in the interaction with the human ACE2 receptor: An in silico analysis. EXCLI J..

[8] Mengist H.M., Kombe A.J.K., Mekonnen D., Abebaw A., Getachew M., Jin T. (2021). Mutations of SARS-CoV-2 spike protein: Implications on immune evasion and vaccine-induced immunity. Semin. Immunol..

[9] Kumar A., Parashar R., Kumar S., Faiq M.A., Kumari C., Kulandhasamy M., Narayan R.K., Jha R.K., Singh H.N., Prasoon P. (2021). Emerging SARS-CoV-2 variants can potentially break set epidemiological barriers in COVID-19. J. Med. Virol..

[10] Pyke A.T., Nair N., van den Hurk A.F., Burtonclay P., Nguyen S., Barcelon J., Kistler C., Schlebusch S., McMahon J., Moore F. (2021). Replication Kinetics of B.1.351 and B.1.1.7 SARS-CoV-2 Variants of Concern Including Assessment of a B.1.1.7 Mutant Carrying a Defective ORF7a Gene. Viruses.

[11] Ahmad L. (2021). Implication of SARS-CoV-2 Immune Escape Spike Variants on Secondary and Vaccine Breakthrough Infections. Front. Immunol..

[12] Galloway S.E., Paul P., MacCannell D.R., Johansson M.A., Brooks J.T., MacNeil A., Slayton R.B., Tong S., Silk B.J., Armstrong G.L. (2021). Emergence of SARS-CoV-2 B.1.1.7 Lineage–United States, 29 December 2020–12 January 2021. Morb. Mortal. Wkly. Rep..

[13] Zhang Y., He X., Zhai J., Ji B., Man V.H., Wang J. (2021). In silico binding profile characterization of SARS-CoV-2 spike protein and its mutants bound to human ACE2 receptor. Brief. Bioinform..

[14] Suleman M., Yousafi Q., Ali J., Ali S.S., Hussain Z., Ali S., Waseem M., Iqbal A., Ahmad S., Khan A. (2021). Bioinformatics analysis of the differences in the binding profile of the wild-type and mutants of the SARS-CoV-2 spike protein variants with the ACE2 receptor. Comput. Biol. Med..

[15] Ortega J.T., Pujol F.H., Jastrzebska B., Rangel H.R. (2021). Mutations in the SARS-CoV-2 spike protein modulate the virus affinity to the human ACE2 receptor, an in silico analysis. EXCLI J..

[16] Hodcroft E.B. (2021). CoVariance: SARS-CoV-2 Mutations and Variants of Interest. https://covariants.org/.

[17] Elbe S., Buckland-Merrett G. (2017). Data, disease and diplomacy: GISAID’s innovative contribution to global health. Glob. Chall..

[18] Walls A.C., Park Y.J., Tortorici M.A., Wall A., McGuire A.T., Veesler D. (2020). Structure, Function, and Antigenicity of the SARS-CoV-2 Spike Glycoprotein. Cell.

[19] Rangel H.R., Ortega J.T., Maksoud S., Pujol F.H., Serrano M.L. (2020). SARS-CoV-2 host tropism: An in silico analysis of the main cellular factors. Virus Res..

[20] Arnold K., Bordoli L., Kopp J., Schwede T. (2006). The SWISS-MODEL workspace: A web-based environment for protein structure homology modelling. Bioinformatics.

[21] Wiederstein M., Sippl M.J. (2007). ProSA-web: Interactive web service for the recognition of errors in three-dimensional structures of proteins. Nucleic Acids Res.

[22] Laskowski R.A., Moss D.S., Thornton J.M. (1993). Main-Chain Bond Lengths and Bond Angles in Protein Structures. J. Mol. Biol..

[23] Ortega J.T., Serrano M.L., Pujol F.H., Rangel H.R. (2020). Unrevealing sequence and structural features of novel coronavirus using in silico approaches: The main protease as molecular target. EXCLI J..

[24] Ortega J.T., Serrano M.L., Jastrzebska B. (2020). Class A G Protein-Coupled Receptor Antagonist Famotidine as a Therapeutic Alternative against SARS-CoV2: An In Silico Analysis. Biomolecules.

[25] Pierce B.G., Wiehe K., Hwang H., Kim B.H., Vreven T., Weng Z. (2014). ZDOCK server: Interactive docking prediction of protein-protein complexes and symmetric multimers. Bioinformatics.

[26] Xue L.C., Rodrigues J.P., Kastritis P.L., Bonvin A.M., Vangone A. (2016). PRODIGY: A web server for predicting the binding affinity of protein-protein complexes. Bioinformatics.

[27] van Zundert G.C.P., Rodrigues J., Trellet M., Schmitz C., Kastritis P.L., Karaca E., Melquiond A.S.J., van Dijk M., de Vries S.J., Bonvin A. (2016). The HADDOCK2.2 Web Server: User-Friendly Integrative Modeling of Biomolecular Complexes. J. Mol. Biol..

[28] Kuriata A., Gierut A.M., Oleniecki T., Ciemny M.P., Kolinski A., Kurcinski M., Kmiecik S. (2018). CABS-flex 2.0: A web server for fast simulations of flexibility of protein structures. Nucleic Acids Res..

[29] Queiros-Reis L., Gomes da Silva P., Goncalves J., Brancale A., Bassetto M., Mesquita J.R. (2021). SARS-CoV-2 Virus-Host Interaction: Currently Available Structures and Implications of Variant Emergence on Infectivity and Immune Response. Int. J. Mol. Sci..

[30] Li T., Han X., Gu C., Guo H., Zhang H., Wang Y., Hu C., Wang K., Liu F., Luo F. (2021). Potent SARS-CoV-2 neutralizing antibodies with protective efficacy against newly emerged mutational variants. Nat. Commun..

[31] Wang P., Nair M.S., Liu L., Iketani S., Luo Y., Guo Y., Wang M., Yu J., Zhang B., Kwong P.D. (2021). Antibody resistance of SARS-CoV-2 variants B.1.351 and B.1.1.7. Nature.

[32] Koehler M., Ray A., Moreira R.A., Juniku B., Poma A.B., Alsteens D. (2021). Molecular insights into receptor binding energetics and neutralization of SARS-CoV-2 variants. Nat. Commun..

[33] Harvey W.T., Carabelli A.M., Jackson B., Gupta R.K., Thomson E.C., Harrison E.M., Ludden C., Reeve R., Rambaut A., Consortium C.-G.U. (2021). SARS-CoV-2 variants, spike mutations and immune escape. Nat. Rev. Microbiol..

[34] Chen C., Boorla V.S., Banerjee D., Chowdhury R., Cavener V.S., Nissly R.H., Gontu A., Boyle N.R., Vandegrift K., Nair M.S. (2021). Computational prediction of the effect of amino acid changes on the binding affinity between SARS-CoV-2 spike RBD and human ACE2. Proc. Natl. Acad. Sci. USA.

[35] Neerukonda S.N., Vassell R., Lusvarghi S., Wang R., Echegaray F., Bentley L., Eakin A.E., Erlandson K.J., Katzelnick L.C., Weiss C.D. (2021). SARS-COV-2 Delta variant displays moderate resistance to neutralizing antibodies and spike protein properties of higher soluble ACE2 sensitivity, enhanced cleavage and fusogenic activity. Viruses.

[36] Lazarevic I., Pravica V., Miljanovic D., Cupic M. (2021). Immune Evasion of SARS-CoV-2 Emerging Variants: What Have We Learnt So Far?. Viruses.

[37] Planas D., Veyer D., Baidaliuk A., Staropoli I., Guivel-Benhassine F., Rajah M.M., Planchais C., Porrot F., Robillard N., Puech J. (2021). Reduced sensitivity of SARS-CoV-2 variant Delta to antibody neutralization. Nature.

[38] Xie X., Liu Y., Liu J., Zhang X., Zou J., Fontes-Garfias C.R., Xia H., Swanson K.A., Cutler M., Cooper D. (2021). Neutralization of SARS-CoV-2 spike 69/70 deletion, E484K and N501Y variants by BNT162b2 vaccine-elicited sera. Nat. Med..

[39] Ewer K.J., Barrett J.R., Belij-Rammerstorfer S., Sharpe H., Makinson R., Morter R., Flaxman A., Wright D., Bellamy D., Bittaye M. (2021). T cell and antibody responses induced by a single dose of ChAdOx1 nCoV-19 (AZD1222) vaccine in a phase 1/2 clinical trial. Nat. Med..

[40] Ravichandran S., Coyle E.M., Klenow L., Tang J., Grubbs G., Liu S., Wang T., Golding H., Khurana S. (2020). Antibody signature induced by SARS-CoV-2 spike protein immunogens in rabbits. Sci. Transl. Med..

[41] Gu Y., Cao J., Zhang X., Gao H., Wang Y., Wang J., He J., Jiang X., Zhang J., Shen G. (2021). Receptome profiling identifies KREMEN1 and ASGR1 as alternative functional receptors of SARS-CoV-2. Cell Res..

[42] Chittum J.E., Sankaranarayanan N.V., O’Hara C.P., Desai U.R. (2021). On the Selectivity of Heparan Sulfate Recognition by SARS-CoV-2 Spike Glycoprotein. ACS Med. Chem. Lett..

[43] Khan A., Mohammad A., Haq I., Nasar M., Ahmad W., Yousafi Q., Suleman M., Ahmad S., Albutti A., Khan T. (2021). Structural-Dynamics and Binding Analysis of RBD from SARS-CoV-2 Variants of Concern (VOCs) and GRP78 Receptor Revealed Basis for Higher Infectivity. Microorganisms.

