# Supplementary material for: Omicron SARS-CoV-2 Variant Spike Protein Shows an Increased Affinity to the Human ACE2 Receptor: An In Silico Analysis
Source: Pathogens. 2021 Dec 31;11(1):45. doi: 10.3390/pathogens11010045 (PMC8779645; doi:10.3390/pathogens11010045)
Supplement: Supplementary file 1 [file pathogens-11-00045-s001.zip › pathogens-1518813-supplementary.pdf]

pathogens

MDPI

Supplementary Information

Omicron SARS-CoV-2 variant spike protein shows an increased affinity to the human ACE2 receptor, an in silico analysis

Joseph Thomas Ortega[1], Beata Jastrzebska[1]*and Hector Rafael Rangel[2]*

[1] Department of Pharmacology, Cleveland Center for Membrane and Structural Biology, School of Medicine, Case Western Reserve University, USA

[2] Laboratorio de Virología Molecular, Centro de Microbiología y Biología Celular, Instituto Venezolano de Investigaciones Científicas, Venezuela

* Hector R. Rangel, Ph.D., Laboratorio de Virología Molecular, Centro de Microbiología y Biología Celular, Instituto Venezolano de Investigaciones Científicas, Apdo 20632, Caracas 1020A, Venezuela; E-mail: hrangel2006@gmail.com

Beata Jastrzebska, Ph.D., Department of Pharmacology, Cleveland Center for Membrane and Structural Biology, School of Medicine, Case Western Reserve University, 10900 Euclid Ave, Cleveland, OH 44106-4965, USA; Phone: 216-368-4631; Fax: 216-368-1300; E-mail: bxj27@case.edu

B.1	319	RVQPTESIVRFPNITNLCPFGEVFNATRFASVYAWNRKRISNCVADYSVLYNSASFSTFK
B.1.617.2	319	RVQPTESIVRFPNITNLCPFGEVFNATRFASVYAWNRKRISNCVADYSVLYNSASFSTFK
B.1.1.529	319	RVQPTESIVRFPNITNLCPFDEVFNATRFASVYAWNRKRISNCVADYSVLYNLAPFFTFK
B.1	379	CYGVSPTKLNDLCFTNVYADSFVIRGDEVRQIAPGQTGKIADYNYKLPDDFTGCVIAWNS
B.1.617.2	379	CYGVSPTKLNDLCFTNVYADSFVIRGDEVRQIAPGQTGKIADYNYKLPDDFTGCVIAWNS
B.1.1.529	379	CYGVSPTKLNDLCFTNVYADSFVIRGDEVRQIAPGQTGNIADYNYKLPDDFTGCVIAWNS
B.1	439	**NNLDSKVGGNYNYLYRLFRKSNLKPFERDISTEIYQAGSTPCNGVEGFNCYFPLQSYGFQ**
B.1.617.2	439	**NNLDSKVGGNYNYRYRLFRKSNLKPFERDISTEIYQAGSKPCNGVEGFNCYFPLQSYGFQ**
B.1.1.529	439	**NKLDSKVSGNYNYLYRLFRKSNLKPFERDISTEIYQAGNKPCNGVAGFNCYFPLRSYSFR**
B.1	499	**PTNGVGYQ**PYRVVVLSFELLHAPATVCGPKK
B.1.617.2	499	**PTNGVGYQ**PYRVVVLSFELLHAPATVCGPKK
B.1.1.529	499	**PTYGVGHQ**PYRVVVLSFELLHAPATVCGPKK

Figure S1. Alignment of a portion of the SARS-CoV-2 spike sequence that includes the receptor-binding domain (RBD) shown in bold. B.1 (Wild Type), B.1.617.2 (Delta), B.1.1.529 (Omicron). Mutations in Delta and Omicron are labeled in blue and red, respectively.

Supplementary Methods.

Protein modeling and computational analysis

The Omicron variant data was retrieved from GISAID [17] and covariants.org webservers (GISAID code WT EPI_ISL_759860, Delta EPE_ISL_3298770, and Omicron EPI_ISL_6699728) on November 26, 2021. Sequence alignment was performed using DNAman (Lynnon Biosoft). The FASTA sequence of the WT SARS-CoV-2 viral spike protein was retrieved from the Uniprot server (sequence number P0DTC2). The Omicron mutated residues were included manually in the FASTA file, and a homology structural model was built using the tools of the SWISS-MODEL modeling server and the DeepView/Swiss-PdbViewer 4.01 software [20]. Then, the model quality was validated via ProSA-web and PROCHECK programs [21,22]. The model was optimized by adding hydrogen atoms and assigning partial charges energy refinement. Followed by molecular dynamic (MD) simulations with NAMD 2.12, as described in Ortega et al. [15] using the CHARMM force field and Gasteiger charges[23,24]. The obtained structure represents the lowest energy frame of the MD simulation. To develop molecular docking the crystal structure of the SARS-CoV-2 spike protein bound to the human ACE2 receptor (PDB code: 6M0J) and the structure of the human ACE2 receptor (PDB code: 1R42) were retrieved from the Protein Data Bank. Then, the binding patterns and affinity estimations for the interaction between the viral spike protein (WT and Omicron variant) and the ACE2 receptor were calculated using two molecular docking approaches. First, protein docking was carried out with Z-Dock [25]. The resulting complexes were analyzed using PROGIDY [26]. Next, the Haddock server was used as a second approach to evaluate the protein docking [27]. Finally, the resulting complexes were evaluated by molecular dynamic simulations. The PDB files were submitted to the CABS-flex server to assess the stability of the spike protein-ACE2 complex, and the parameters were adjusted as a default [28]. The MD simulation data were analyzed according to root-mean-square deviation (RMSF).
